# Estimated preventive dose of racemic ketamine for shivering and pruritus prophylaxis in cesarean delivery: a Monte Carlo simulation guided network meta-analysis

**DOI:** 10.3389/fphar.2026.1751842

**Published:** 2026-02-04

**Authors:** Wei-Long Wang, Jin Zhou, Yi Cai, Min-Zhu Zheng, Kai-Yu Chen, Ying Hu, Xin Men, Jian-Liang Sun, Xu Qiu, Zhen-Feng Zhou

**Affiliations:** 1 The Fourth Clinical Medical College, Zhejiang Chinese Medicine University, Hangzhou, China; 2 Department of Anesthesiology, Hangzhou First People’s Hospital, Hangzhou, China; 3 Department of Delivery Center, Hangzhou Women’s Hospital (Hangzhou Maternity and Child Healthcare Hospital, Hangzhou First People’s Hospital Qianjiang New City Campus, Zhejiang Chinese Medical University), Hangzhou, China; 4 Department of Anesthesiology, Hangzhou Women’s Hospital (Hangzhou Maternity and Child Healthcare Hospital, Hangzhou First People’s Hospital Qianjiang New City Campus, Zhejiang Chinese Medical University), Hangzhou, China

**Keywords:** adverse effects, dose-responses, ketamine, Monte Carlo simulation, network meta analysis, pruritus, shivering

## Abstract

**Background:**

The use of ketamine and esketamine in cesarean delivery is limited by their dose-dependent adverse effects. This study aimed to precisely quantify the dose-response relationships for the prevention of shivering and pruritus and to determine the associated risk of neuropsychiatric side effects, thereby defining its therapeutic window.

**Methods:**

A systematic review and network meta-analysis were conducted. We searched databases for randomized controlled trials (RCTs) evaluating a single intravenous bolus of ketamine or esketamine during cesarean delivery under neuraxial anesthesia. Study quality was assessed using the Cochrane RoB 2 tool. We integrated traditional and network meta-analysis with logistic regression, Monte Carlo simulation, and polynomial regression to establish continuous dose-response models and calculate key dose parameters (ED_50_, ED_95_).

**Results:**

25 studies(3,842 participants) were included. The ED_50_ for preventing pruritus and shivering were 0.122 mg/kg (95% CI, 0.087–0.164) and 0.329 mg/kg (95% CI, 0.260–0.412), respectively. However, at doses of 0.273 mg/kg (ED_50_) and 0.761 mg/kg (ED_95_), 50% and 95% of parturients, respectively, experienced subjective side effects.

**Conclusion:**

The benefits of low-dose ketamine (≈0.12 mg/kg) for pruritus prophylaxis clearly outweigh its risks. In contrast, the dose required for shivering prevention (≈0.33 mg/kg) falls within the range where side effects become common, resulting in a narrow therapeutic window. This study provides critical dose-finding evidence for individualized, goal-directed use of ketamine in cesarean delivery.

**Systematic Review Registration:**

https://www.crd.york.ac.uk/PROSPERO/view/CRD420251073122, identifier CRD420251073122.

## Highlights

- This study aimed to precisely quantify the dose-response relationships for the prevention of shivering and pruritus.
- The ED_50_ for ketamine in preventing pruritus is 0.122 mg/kg (95%CI, 0.087–0.164), while the ED_50_ for reducing shivering is 0.329 mg/kg(95%CI, 0.260–0.412).
- At doses of 0.273 mg/kg(ED_50_, 95%CI, 0.223–0.314) and 0.761 mg/kg(ED_95_, 95%CI, 0.669–0.880), 50% and 95% of women experience ≥1 neuropsychiatric adverse effect, respectively.
- This study provides critical dose-finding evidence for individualized, goal-directed use of ketamine in cesarean delivery.

## Introduction

During cesarean delivery under spinal anesthesia, maintaining maternal comfort while the patient remains awake presents a unique clinical challenge. Beyond pain, shivering and pruritus are two common complications that significantly impair the patient’s experience (Carvalho et al., 2005).

Intraoperative shivering not only increases maternal discomfort and anxiety but can also interfere with vital sign monitoring, elevate metabolic oxygen consumption, and has been associated with a heightened risk of postoperative infection. Although ketamine has been shown to possess some prophylactic efficacy against shivering (Jouryabi et al., 2021; Lema et al., 2017; Yang et al., 2024), its optimal dosing regimen and the balance between its benefits and adverse effects remain unclear.

Neuraxial opioids are a standard component of analgesia for cesarean delivery, but they carry a high incidence of inducing pruritus, which markedly compromises maternal comfort and satisfaction. While recent years have seen considerable discussion on the mechanisms and prevention of opioid-induced pruritus (Singh et al., 2023; Qin et al., 2025), the role of ketamine in this context lacks systematic investigation. Its potential value and the appropriate dosage for preventing pruritus have yet to be established.

Additionally, there are still two key issues regarding the clinical application of ketamine: Firstly, it may cause neurological and psychological adverse reactions such as hallucinations, dizziness, and nystagmus, which can affect the experience and safety of the mother during childbirth; Secondly, there is currently no unified dosing protocol, and the dose-dependent relationship between efficacy and adverse reactions has not been clearly defined. Therefore, while striving for therapeutic effects, systematically assessing the dose-related risks is the prerequisite for achieving individualized medication and optimizing clinical decisions (Rid et al., 2010; Platt et al., 2014).

Therefore, this study aimed to quantitatively determine the dose-response relationships of ketamine/esketamine for preventing shivering and pruritus during cesarean delivery, and to define the concurrent risk of dose-dependent subjective adverse effects (e.g., hallucinations, dizziness), thereby identifying the optimal therapeutic dose range.

## Methods

### Protocol and registration

The protocol for this systematic review and network meta-analysis was prospectively registered with PROSPERO (Registration Number: CRD420251073122). The design and reporting of this systematic review adhere to the Preferred Reporting Items for Systematic Reviews and Meta-Analyses (PRISMA) guidelines (Page et al., 2021). The completed PRISMA checklist is provided as Supplementary Table S1. This study followed TITAN guidelines 2025 (Agha et al., 2025) and we did not use any AI tools during the entire research process.

### Literature search

We systematically searched three peer-reviewed databases—Cochrane Library, PubMed, and Embase—from their inception to 25 April 2025. Two investigators independently executed the search using predefined terms, including “Ketamine,” “Cesarean,” and “randomized controlled trials” combined with Boolean operators. Full search syntax for each database is provided in Supplementary Table S2.

### Study selection

Two investigators independently screened titles and abstracts for relevance using predefined inclusion criteria. Eligible studies were required to meet the following criteria: (1) Population: Women undergoing cesarean delivery under neuraxial (spinal or combined spinal-epidural) anesthesia. (2) Intervention: A single intravenous bolus of ketamine or esketamine. (3) Comparator: Placebo (saline) or no intervention. (4) Outcomes: Reporting of data on at least one of the pre-specified efficacy or safety outcomes for this meta-analysis (listed in the ‘Outcomes’ section below). (5) Study design: Randomized controlled trials (RCTs).

Exclusion criteria comprised: (1) studies where ketamine was not administered as a single intravenous bolus; (2) studies combining ketamine with other active agents without a separate ketamine-only group; (3) cesarean sections performed under general anesthesia; (4) animal studies; (5) Systematic reviews and meta-analyses, conference abstracts, trial registry records and retracted articles were also excluded; (6) non-English language studies.

Subsequently, the same investigators rigorously assessed the full-text articles to confirm eligibility and extract data using a standardized form. Any discrepancies or conflicts between the two investigators during the screening or data extraction phases were resolved through discussion and consensus. If consensus could not be reached, a third senior investigator (Zhen-feng ZHOU) was consulted for arbitration.

### Data extraction and outcomes

A standardized data extraction form was used. The following items were extracted from each included study by two independent investigators: (1) Study and Participant Characteristics: first author, publication year, country, and participant demographics including sample size per group, mean age, body mass index (BMI), and gestational age. (2) Intervention details: specific drug (ketamine or esketamine), dose (in mg/kg), and timing of administration (relative to spinal block or surgery). (3) Outcome data: For each pre-specified efficacy (shivering, pruritus) and safety outcome (e.g., hallucinations, dizziness, nausea/vomiting, hypotension), the number of events and the total number of participants assessed in each treatment group were extracted. (4) Methodological Data: Key information required for the assessment of risk of bias using the Cochrane RoB 2 tool. (5) Other Notes: Details of anesthesia technique, intraspinal drug regimen, and any other relevant comments.

Identified efficacy outcomes included shivering and pruritus, while safety outcomes encompassed nystagmus, diplopia, hallucinations, drowsiness, dizziness, headache, hypotension, nausea, and vomiting. Definitions for all outcomes are provided in Supplementary Appendix 1.

### Data synthesis and analysis

Data from individual randomized controlled trials were extracted through Microsoft Office Excel and analyzed by R version 4.4.0 (R Foundation for Statistical Computing, Vienna, Austria; www.R-project.org) and RevMan version 5.3 meta-analysis (Copenhagen: Nordic Cochrane Center, Cochrane Collaborative Organization, 2014).

### Dose equivalent conversion

Based on evidence that the anesthetic potency of esketamine is approximately twice that of racemic ketamine (Mion and Himmelseher, 2024), dose normalization was performed to ensure comparability across studies. All reported esketamine doses were multiplied by a factor of two to yield ketamine-equivalent doses. Subsequent analyses—including dose–response modeling and Monte Carlo simulations—were conducted using these standardized doses to ensure consistent pharmacologic potency across the dataset.

### Analytical approach

This study aimed to quantify ED_95_ and ED_50_ values for ketamine-induced any subjective discomfort symptoms (hallucinations, diplopia, dizziness, headache, drowsiness) during cesarean delivery. Study-level data (sample sizes and event counts per outcome at specific doses) were utilized to simulate individual patient-level data. Logistic regression with continuous dose modeling established dose-response relationships for each endpoint, implemented in R 4.4.0 using the glm() function with dose-response visualizations generated via ggplot2.

This study employed an integrated approach combining Monte Carlo simulation and polynomial regression to determine ED_95_ and ED_50_. Computer simulations generated 1,001,000 virtual subjects equally distributed across 1001 dose groups (1000 subjects per group). For each dose group, the mean incidence rate and corresponding 95% confidence interval derived from logistic regression models informed the generation of normally distributed datasets via the rnorm() function. A single value was randomly sampled from each distribution to represent the theoretical event incidence rate for the respective dose group. Sampled values below 0 were truncated to 0, while values exceeding 1 were truncated to 1. The truncated incidence rates subsequently guided probabilistic sampling of binary event outcomes (occurrence/non-occurrence). This sampling cycle was iteratively executed, ultimately yielding 1,001,000 simulated parturients per study endpoint. A fixed random seed (Seed = 20250802) ensured procedural reproducibility throughout randomization.

Polynomial regression modeling quantified dose-dependent risk levels by fitting relationships between ketamine dose and subjective discomfort symptom incidence. Concurrently, polynomial regression simulations estimated preventive efficacy incidence rates at each dose point. Simulated data were used to calculate the dose and standard deviation for a 50% reduction in the incidence of each outcome of preventive treatment.

### Quality assessment of the included studies

Methodological rigour was assessed using the Cochrane RoB 2.0 tool (www.riskofbias.info) to evaluate bias risk in randomised controlled trials (Higgins et al., 2011). Each study was categorised as having low risk, some concerns, or high risk of bias through structured appraisal across five domains: (1) randomisation process, (2) deviations from intended interventions, (3) missing outcome data, (4) measurement of outcomes, and (5) selection of reported results. Domain-specific signalling questions (response options: Yes/Probably yes/Probably no/No/No information) guided critical evaluation (Akl et al., 2012). Final risk classifications were derived by synthesising domain-level judgements in accordance with the RoB 2.0 algorithm. Five reviewers independently conducted methodological evaluations, with each study assessed by at least two reviewers. Discrepancies were resolved through iterative re-evaluation and team discussion until consensus was achieved, following a predefined adjudication protocol. Attempts were made to contact the authors to obtain missing data, and studies with incomplete data were excluded when comparing specific outcomes.

### Core assumptions of NMA

The validity of Network Meta-Analysis (NMA) results rests on three core assumptions: First, homogeneity: Studies comparing the same treatments were comparable (Song et al., 2003; Salanti, 2012). The second is similarity, which extends homogeneity to networks: All included studies were comparable (Dias et al., 2013a; Dias et al., 2013b). We evaluated the distribution of potential effect modifiers across treatment comparisons using a network plot of clinical characteristics.

The third assumption is consistency: estimates using direct and indirect evidence are similar (Shih and Tu, 2021; Tu, 2015; White et al., 2012; Yu-Kang, 2016). For each comparison, direct odds ratios (ORs) derived from head-to-head testing and indirect ORs estimated by network pathways were calculated. The difference between the sources of evidence was quantified by the ratio of odds ratio (ROR), defined as: ROR = direct OR/indirect OR. Agreement was statistically confirmed if the 95% confidence interval of ROR included a null value of 1(P > 0.05, two-sided).

### Sensitivity analyses

To assess the robustness of primary meta-analysis results, sensitivity analyses were performed for outcomes exhibiting substantial heterogeneity (*I* ^2^ ≥ 50%) and Cochran’s Q test (using a significance threshold of p < 0.10). This involved the leave-one-out method, where each study was sequentially excluded to evaluate its individual influence on the pooled effect estimate and heterogeneity.

When heterogeneity persisted or was anticipated based on clinical rationale, pre-specified subgroup analyses were conducted to explore potential sources of variability. Factors considered included, geographic region, type of intraspinal anesthetic/adjuvant, and timing of ketamine administration, ketamine or esketamine used. Subgroup differences were examined by inspecting the overlap of 95% CIs and assessing the reduction in residual heterogeneity within subgroups.

### Assessing the methodological quality of systematic review

The methodological quality of the included systematic reviews was critically appraised using the AMSTAR 2 (A MeaSurement Tool to Assess systematic Reviews 2) checklist. This instrument provides a comprehensive and reliable assessment of key aspects of review conduct, including the adequacy of the search strategy, risk of bias evaluation, and appropriateness of meta-analytical methods. The overall confidence in the results of each review was graded accordingly (Shea et al., 2017).

## Result

### Literature search

**FIGURE 1:**
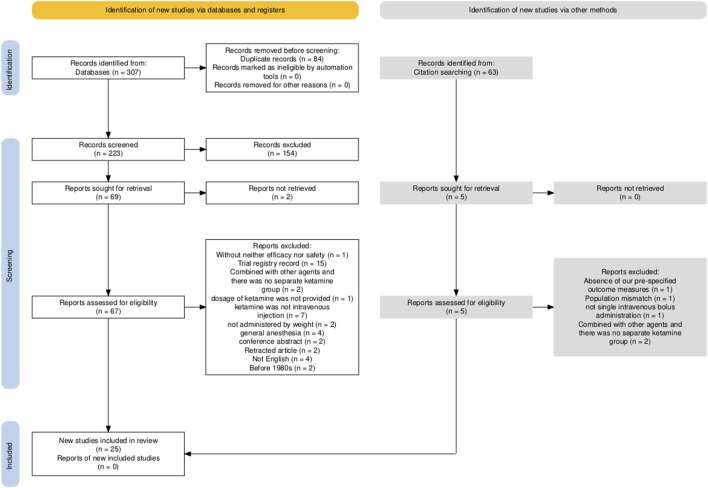
The PRISMA flow chart.

**FIGURE 2:**
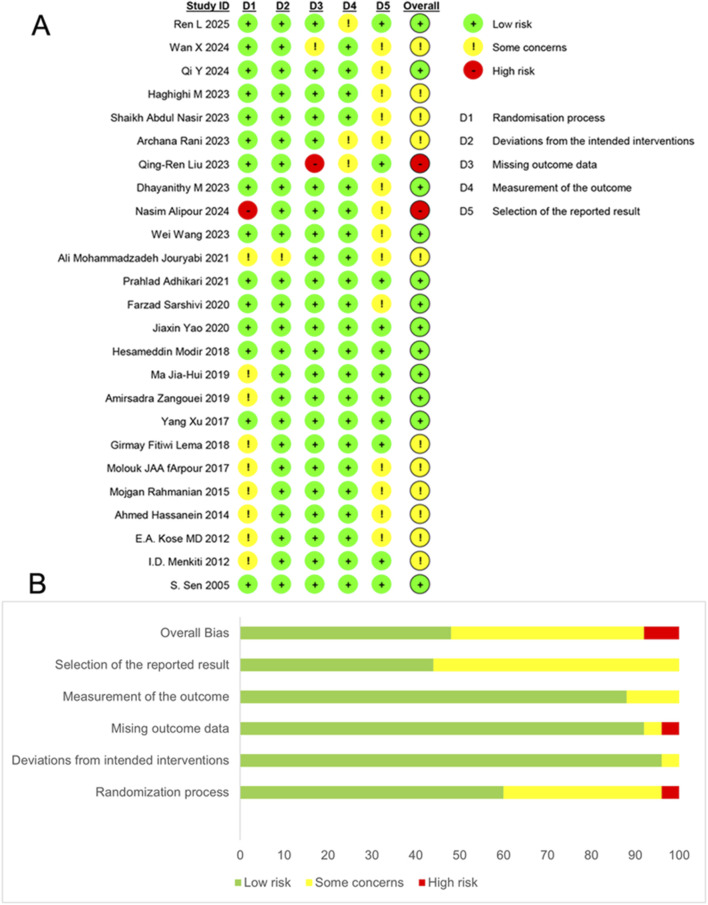
Risk of bias assessments. **(A)**, Cochrane-style risk of bias figures. **(B)**, plot of the percentage of risk of bias assessments at each level of risk of bias per domain.

Our initial search identified 223 articles classified as RCTs. Following screening of titles and abstracts, 69 articles underwent full-text review. Ultimately, 25 articles (Jouryabi et al., 2021; Ren et al., 2025; Sen et al., 2005; Menkiti et al., 2012; Kose et al., 2013; Hassanein and Mahmoud, 2015; Rahmanian et al., 2015; Jaafarpour et al., 2017; Lema et al., 2017; Xu et al., 2017; Zangouei et al., 2019a; Ma et al., 2019; Modir et al., 2019; Yao et al., 2020; Sarshivi et al., 2020; Adhikari et al., 2021; Wang et al., 2023; Liu et al., 2023; Rani et al., 2023; Nasir et al., 2023; Dhayanithy et al., 2023; Haghighi et al., 2023; Alipour et al., 2024; Qi et al., 2024; Wan et al., 2024) involving 3842 participants were included in this meta-analysis (Figure 1) (Haddaway et al., 2022). Most of the included articles were reported nearly 10 years and mainly come from Asia especially from Iran and China. Twenty articles (Jouryabi et al., 2021; Sen et al., 2005; Menkiti et al., 2012; Kose et al., 2013; Hassanein and Mahmoud, 2015; Rahmanian et al., 2015; Jaafarpour et al., 2017; Lema et al., 2017; Xu et al., 2017; Zangouei et al., 2019a; Modir et al., 2019; Yao et al., 2020; Sarshivi et al., 2020; Adhikari et al., 2021; Rani et al., 2023; Nasir et al., 2023; Dhayanithy et al., 2023; Haghighi et al., 2023; Alipour et al., 2024) reported the use of ketamine and five articles (Ren et al., 2025; Wang et al., 2023; Liu et al., 2023; Qi et al., 2024; Wan et al., 2024) reported the use of esketamine. The data on the primary outcomes of shivering (Jouryabi et al., 2021; Menkiti et al., 2012; Kose et al., 2013; Rahmanian et al., 2015; Jaafarpour et al., 2017; Lema et al., 2017; Sarshivi et al., 2020; Adhikari et al., 2021; Dhayanithy et al., 2023) and pruritus (Sen et al., 2005; Jaafarpour et al., 2017; Zangouei et al., 2019a; Rani et al., 2023) were only available from ketamine trials. However, for all other outcomes, data were available from both ketamine and esketamine trials with the application of the dose conversion factor. The characteristic of population such as age, body mass index (BMI) and gestational age were performed in Table 1. We assessed the risk of bias for each individual study and the overall risk of bias (Figure 2). Nearly half of the articles (12/25) (Ren et al., 2025; Sen et al., 2005; Xu et al., 2017; Zangouei et al., 2019a; Ma et al., 2019; Modir et al., 2019; Yao et al., 2020; Sarshivi et al., 2020; Adhikari et al., 2021; Wang et al., 2023; Dhayanithy et al., 2023; Qi et al., 2024) were judged to be at low risk of bias. The number of articles rated as having some concerns regarding bias (11/25) (Jouryabi et al., 2021; Menkiti et al., 2012; Kose et al., 2013; Hassanein and Mahmoud, 2015; Rahmanian et al., 2015; Jaafarpour et al., 2017; Lema et al., 2017; Rani et al., 2023; Nasir et al., 2023; Haghighi et al., 2023; Wan et al., 2024) was second only to those at low risk. Two articles (Liu et al., 2023; Alipour et al., 2024) were assessed as having a high risk of bias. The similarity assumption was satisfied by the evaluation of baseline characteristics (age, BMI, gestational age), and the dot plot (Supplementary Figure S1) confirmed the stable distribution of baseline data in each study.

**TABLE 1:** Characteristics of 25 randomised controlled trials included in meta-analyses of Ketamine for cesarean delivery.

Characteristics	No (%) of trials unless stated otherwise
Year of publication	
Up to 2010	1 (4)
2011∼2015	4 (16)
2016∼2020	8 (32)
2021∼2025	12 (48)
Country area	
Europe	2 (8)
Asia	20 (80)
Africa	3 (12)
Age (years), mean ± SD^a^	28.89 ± 4.64
BMI (kg·m^-2^), mean ± SD^a^	27.92 ± 4.27
Gestational age (week), mean ± SD^a^	38.36 ± 1.26
Methods of anesthesia	
CSEA^b^	3 (12)
SA^c^	21 (84)
Not mentioned	1 (4)
Intravenous	
Esketamine	5 (20)
Ketamine	20 (80)
Intraspinal drugs	
Bupivacaine	16 (64)
Bupivacaine + opioids	3 (12)
Ropivacaine	2 (8)
Ropivacaine + opioids	2 (8)
Not mentioned	2 (8)
Timing of ketamine administration	
Not mentioned	1 (4)
Before spinal anesthesia	1 (4)
spinal anesthesia---surgical incision	8(32)
surgical incision-----fetal extraction	0 (0)
After fetal extraction	
Immediately	1 (4)
5 min	5 (20)
10 min	3 (12)
20 min	1 (4)
No specific	5 (20)

^a^ SD, standard deviation.

^b^ CSEA, Combined spinal-epidural anesthesia.

^c^ SA, spinal anesthesia.

### Network meta analysis

**FIGURE 3:**
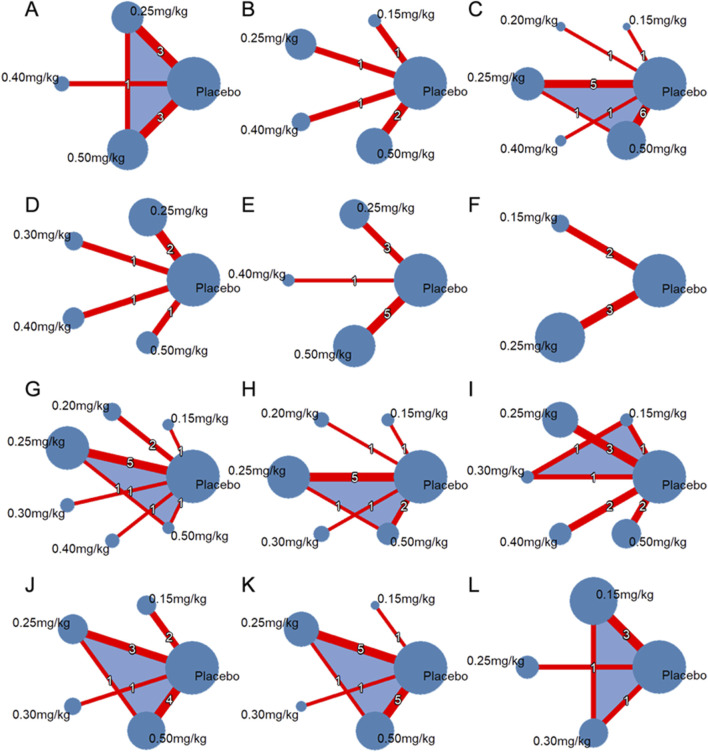
Network maps of doses of ketamine for: **(A)** nystagmus; **(B)** diplopia; **(C)** hallucinations; **(D)** drowsiness; **(E)** dizziness; **(F)** headache; **(G)** hypotension; **(H)** shivering; **(I)** nausea or vomiting; **(J)** nausea; **(K)** vomiting; **(L)** pruritus.

Evidence Network plots(Figure 3) were constructed to visualize direct comparative relationships between interventions. The nodes represent treatments, and the lines represent randomized controlled trials for which direct comparisons exist.

Direct comparison for the 0.20 and 0.30 mg/kg dose groups was not feasible due to insufficient data.

### Dose of 0.15 mg/kg ketamine versus placebo

The different studies for each direct comparison were integrated using a random effects model. In direct comparisons, a dose of 0.15 mg/kg, including 3 studies (Sen et al., 2005; Zangouei et al., 2019a; Rani et al., 2023) with 196 participants ketamine was associated with a significantly reduced risk of pruritus (OR = 0.25, 95% CI: 0.10–0.59; *P* = 0.002; *I* ^2^ < 0.001%) (Supplementary Figure S2A).

No significant association was shown with nausea (Supplementary Figure S2B) or headache (Supplementary Figure S2C) both with 116 participants from 2 studies (Sen et al., 2005; Menkiti et al., 2012).

Because of limited studies with a dose of 0.15 mg/kg ketamine regard to other outcomes for analyze.

### Dose of 0.25 mg/kg ketamine versus placebo

At the 0.25 mg/kg dose, the risks of nystagmus (Jouryabi et al., 2021; Kose et al., 2013; Haghighi et al., 2023) (OR = 24.81; 524 participants; Supplementary Figure S3A), hallucinations (Jouryabi et al., 2021; Kose et al., 2013; Yao et al., 2020; Lavelle and MacIomhair, 1975; Rahmanian et al., 2015) (OR = 11.68; 992 participants; Supplementary Figure S3B), drowsiness (Xu et al., 2017; Jaafarpour et al., 2017) (OR = 16.71; 371 participants; Supplementary Figure S3C), dizziness (Xu et al., 2017; Yao et al., 2020; Jaafarpour et al., 2017) (OR = 20.39; 679 participants; Supplementary Figure S3D), and headache (Jouryabi et al., 2021; Xu et al., 2017; Yao et al., 2020) (OR = 4.08; 887 participants; Supplementary Figure S3E) were significantly higher compared with placebo (all *P* < 0.05; Table 2).

**TABLE 2:** Direct comparison of each dose group versus placebo.

Analyzable ending	OR(95%CI)	*P* value	*I* ^2^(%)	*P* value of *I* ^2^
Efficacy				
0.15 mg/kg				
Pruritus	0.25(0.10, 0.59)	0.002	<0.001	0.86
Nausea	0.46(0.09, 2.27)	0.34	<0.001	0.50
0.25 mg/kg				
Shivering^a^	0.39(0.19, 0.81)	0.01	63	0.03
Nausea/Vomiting	0.44(0.29, 0.66)	<0.0001	<0.001	0.56
Nausea^a^	0.48(0.16, 1.41)	0.18	65	0.06
Vomiting^a^	0.90(0.29, 2.81)	0.85	69	0.01
0.40 mg/kg				
Nausea/vomiting	0.49(0.26, 0.93)	0.03	21	0.28
0.50 mg/kg				
Nausea^a^	0.76(0.34, 1.70)	0.50	65	0.03
Vomiting^a^	1.60(1.14, 2.25)	0.006	59	0.04
Safety				
0.15 mg/kg				
Headache	0.47(0.11, 2.06)	0.32	<0.001	0.54
0.25 mg/kg				
Nystagmus	24.81(4.70, 131.03)	0.0002	<0.001	0.61
Hallucination^a^	11.68(1.62, 84.40)	0.01	68	0.03
Drowsiness	16.71(3.79, 73.67)	0.0002	57	0.13
Dizziness^a^	20.39(5.41, 76.77)	<0.0001	78	0.01
Headache	4.08(1.82, 9.18)	0.0007	7	0.34
Hypotension	0.51(0.36, 0.74)	0.0004	<0.001	0.40
0.50 mg/kg				
Nystagmus^a^	193.32(8.14, 4593.64)	0.001	71	0.03
Hallucination	7.96(2.08, 30.43)	0.002	45	0.11
Dizziness^a^	11.18(1.46, 85.84)	0.02	89	<0.0001

0.20/0.30 mg/kg dose groups was not feasible due to insufficient data.

^a^ showed significant heterogeneity and need to sensitivity analysis.

Abbreviation: OR, odds ratio; CI, confidence interval.

Conversely, the risks of hypotension (Jouryabi et al., 2021; Menkiti et al., 2012; Adhikari et al., 2021; Rahmanian et al., 2015; Fakheri et al., 2023) (OR = 0.51; 764 participants; Supplementary Figure S3F), shivering (Jouryabi et al., 2021; Kose et al., 2013; Adhikari et al., 2021; Rahmanian et al., 2015; Jaafarpour et al., 2017) (OR = 0.39; 600 participants; Supplementary Figure S3G), and nausea/vomiting (Jouryabi et al., 2021; Adhikari et al., 2021; Fakheri et al., 2023) (OR = 0.44; 554 participants; Supplementary Figure S3H) were significantly lower than with placebo (all *P* < 0.05). No significant association was observed for nausea (Kose et al., 2013; Rahmanian et al., 2015; Jaafarpour et al., 2017) (266 participants; Supplementary Figure S3I) or vomiting (Kose et al., 2013; Xu et al., 2017; Yao et al., 2020; Rahmanian et al., 2015; Jaafarpour et al., 2017) (899 participants; Supplementary Figure S3J) (all *P* > 0.05; Table 2).

Significant heterogeneity was detected for hallucinations, dizziness, shivering, nausea, and vomiting (*I* ^2^ > 50%, *P* < 0.10), warranting sensitivity analyses (Supplementary Table S3).

### Dose of 0.40 mg/kg ketamine versus placebo

At the 0.40 mg/kg dose (Hassanein and Mahmoud, 2015; Wang et al., 2023; Qi et al., 2024), ketamine significantly reduced the risk of nausea/vomiting compared with placebo (OR = 0.49, *P* = 0.03; 325 participants) (Table 2; Supplementary Figure S4), with low heterogeneity (*I* ^2^ = 21%, *P* = 0.21).

### Dose of 0.50 mg/kg ketamine versus placebo

At the 0.50 mg/kg dose, the risks of nystagmus (Kose et al., 2013; Wan et al., 2024; Ma et al., 2019) (OR = 193.32; 843 participants; Supplementary Figure S5A), hallucinations (Ren et al., 2025; Kose et al., 2013; Liu et al., 2023; Alipour et al., 2024; Wan et al., 2024; Ma et al., 2019) (OR = 7.96; 1336 participants; Supplementary Figure S5B), dizziness (Ren et al., 2025; Liu et al., 2023; Dhayanithy et al., 2023; Wan et al., 2024; Ma et al., 2019) (OR = 11.18; 1322 participants; Supplementary Figure S5C), and vomiting (Kose et al., 2013; Liu et al., 2023; Dhayanithy et al., 2023; Wan et al., 2024; Ma et al., 2019) (OR = 1.60; 1074 participants; Supplementary Figure S5D) were significantly higher than with placebo (all *P* < 0.05; Table 2). No significant difference was observed for nausea (4 studies (Kose et al., 2013; Liu et al., 2023; Dhayanithy et al., 2023; Wan et al., 2024; Ma et al., 2019), 420 participants; Supplementary Figure S5E).

Significant heterogeneity was observed for most outcomes except hallucinations (*I* ^2^ range, 59%–89%; Table 2), prompting further sensitivity analyses (Supplementary Table S3).

### Sensitivity analysis

Sensitivity analyses were performed for outcomes with substantial heterogeneity (*I* ^2^ > 50%, *P* < 0.10) using sequential study exclusion. The overall effects of ketamine versus placebo across dose groups were generally robust, though instability was noted for several outcomes (Supplementary Table S3).

At the 0.25 mg/kg dose, the pooled effect estimates for hallucinations (after excluding Rahmanian et al. (2015): OR = 27.26, 95% CI: 5.25–141.68) and shivering (after excluding Jaafarpour et al. (2017): OR = 0.39, 95% CI: 0.19–0.81) remained statistically significant, confirming the robustness of these associations. However, the relationship between ketamine and nausea became significant following the exclusion of Rahmanian et al. (2015) (from OR = 0.48, 95% CI: 0.16–1.41, *P* = 0.18 to OR = 0.27, 95% CI: 0.10–0.70, *P* = 0.007), indicating sensitivity to the inclusion of specific studies. For dizziness (*I* ^2^ = 78%) and vomiting (*I* ^2^ = 69%), no definitive source of heterogeneity was identified.

In subgroup analyses of the 0.25 mg/kg dose, geographic region (Chinese vs. non-Chinese) appeared to contribute to the heterogeneity in vomiting. The risk of vomiting differed significantly between ketamine and placebo groups in both Chinese (OR = 3.69, 95% CI: 1.19–11.49, *P* = 0.02; Supplementary Figure S6A) and non-Chinese populations (OR = 0.42, 95% CI: 0.23–0.78, *P* = 0.006; Supplementary Figure S6B), with no residual heterogeneity (*I* ^2^ < 0.0001%, *P* > 0.05). Nonetheless, substantial heterogeneity persisted for dizziness.

At the 0.50 mg/kg dose, the pooled effects for nystagmus (excluding any single study) and nausea remained non-significant across all sensitivity analyses, supporting their stability. Conversely, the association with vomiting lost statistical significance upon exclusion of Ma Jia-Hui 2019 (Kose et al., 2013; Wan et al., 2024; Ma et al., 2019) (from OR = 1.60, 95% CI: 1.14–2.25, *P* = 0.006 to OR = 1.02, 95% CI: 0.61–1.69, *P* = 0.94), indicating limited robustness. No clear heterogeneity source was identified for dizziness (*I* ^2^ = 89%). Subgroup analyses further showed that variations in intraspinal administration type, concomitant intraspinal opioid use, timing of ketamine administration, and specific drug regimens did not explain the observed heterogeneity (Supplementary Table S4).

Comparison of direct and indirect evidence (based on the match tables, structure) was feasible for only two dose pairs: 0.25 mg/kg vs. 0.50 mg/kg and 0.15 mg/kg vs. 0.30 mg/kg. For all assessed adverse events (hypotension, nausea, shiver, hallucination, vomiting, pruritus, nystagmus), no significant inconsistency was found between direct and indirect evidence but with high wide 95%CI increasing their inaccuracy, as indicated by the ratio of odds ratios (ROR) 95% confidence intervals (CIs) encompassing 1 (*P* > 0.05). The specific odds ratios (ORs) and RORs are summarized in Supplementary Table S5.

### Logistic regression

We modeled multiple indicators (hallucinations, diplopia, dizziness, headache, drowsiness, nystagmus, hypertension, shiver, pruritus) using a logistic regression model to determine the response relationship between them and the dose, and provided a 95% confidence interval (Supplementary Figure S7). This step was taken to ensure the subsequent Monte Carlo simulation.

### Monte Carlo simulation and polynomial regression

**FIGURE 4:**
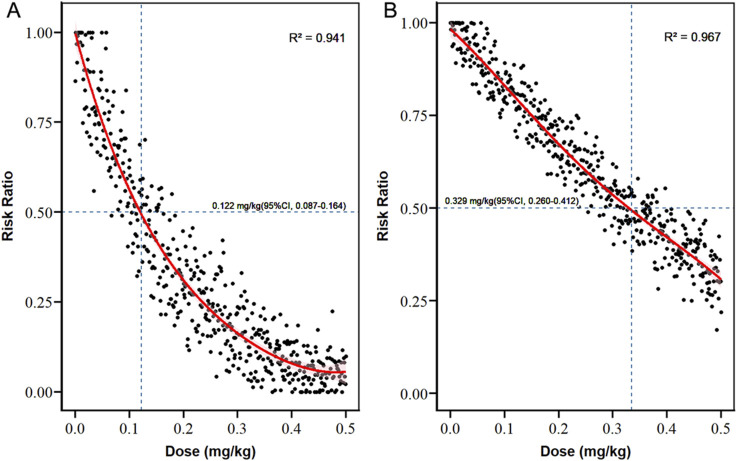
Dose-response curves for the risk of pruritus and shivering. **(A)** pruritus; **(B)** shivering.

We also calculated the predicted dose at a RR of 0.5 for shiver and pruritus, as compared with placebo, which was defined as a halving of the incidence. When 0.122 mg/kg(95%CI, 0.087–0.164) ketamine was used, the incidence of pruritus was reduced to half of the baseline value (Figure 4A; Table 3). When ketamine was used at 0.329 mg/kg(95%CI, 0.260–0.412), the incidence of shivering was reduced to half the baseline value (Figure 4B; Table 3). Polynomial generalized linear regression models were used to fit the RR for shivering (R^2^ = 0.967) and pruritus (R^2^ = 0.941) and the relationship between dose.

**TABLE 3:** Results of Monte Carlo simulation for estimated effect dose.

Outcomes	Estimated effect dose	Estimated 95%CI
Prevention		
Pruritus, ED_50_	0.122	0.087–0.164
Shivering, ED_50_	0.329	0.260–0.412
Subject discomfort		
ED_50_	0.273	0.223–0.314
ED_95_	0.761	0.669–0.880

All results are mixed population model-based estimates and are presented as ED_50_ or ED_95_ with doses in milligrams per kilogram of body weight(mg/kg).

Abbreviation: CI, confidence interval.

**FIGURE 5:**
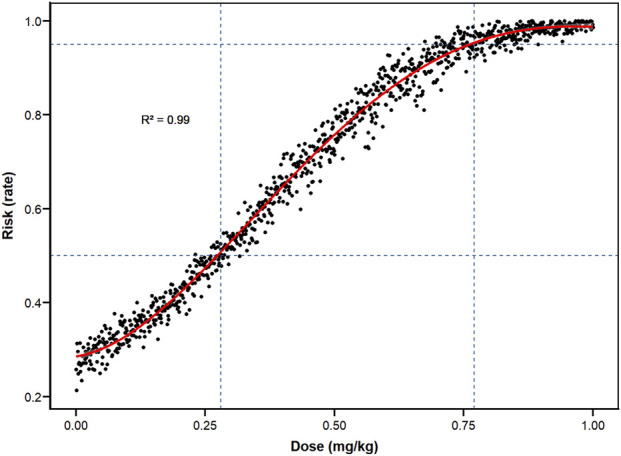
Dose-response curve for either subjective discomfort symptom.

However, it was calculated that when using 0.273 mg/kg(ED_50_, 95%CI, 0.223–0.314) and 0.761 mg/kg(ED_95_, 95%CI, 0.669–0.880) ketamine, respectively, 50% of women and 95% of women experienced any kind of subjective discomfort (hallucinations, diplopia, dizziness, headache or drowsiness). And a polynomial regression curve was plotted (Figure 5; Table 3).

### Sensitivity analysis for simulation in asian populations

To assess potential geographic bias, an additional sensitivity analysis was performed for Asian populations (Supplementary Table S6). The recalibrated model estimated that ketamine doses of 0.128 mg/kg (95% CI: 0.089–0.174) and 0.413 mg/kg (95% CI: 0.302–0.553) reduced the incidences of pruritus and shivering to half of the baseline level respectively-values consistent with the primary model.

Within this subgroup, the estimated doses producing subjective discomfort in 50% and 95% of participants were 0.270 mg/kg (ED_50_, 95% CI: 0.230–0.313) and 0.719 mg/kg (ED_95_, 95% CI: 0.635–0.825), respectively, aligning closely with the overall analysis.

## Discussion

By integrating a multi-stage methodological framework—including traditional meta-analysis, network meta-analysis, logistic regression modeling, Monte Carlo simulation, and polynomial regression—this study comprehensively characterized the dose–response relationship of ketamine in cesarean delivery under spinal anesthesia. We found that the ED_50_ of ketamine for preventing pruritus and reducing shivering were 0.122 mg/kg (95%CI, 0.087–0.164) and 0.329 mg/kg (95%CI, 0.260–0.412), respectively. However, when the ketamine dose increased to 0.273 mg/kg (95%CI, 0.223–0.314) and 0.761 mg/kg (95%CI, 0.669–0.880), 50% and 95% of women, respectively, experienced at least one subjective discomfort (hallucinations, diplopia, dizziness, headache, or drowsiness), which might impaire overall maternal experience. It must be emphasized that although our systematic review protocol included both ketamine and esketamine, no data on the primary outcomes (shivering or pruritus) were available from the esketamine studies. Therefore, dose-response modeling, Monte Carlo simulations, and ED_50_ estimates presented for shivering or pruritus are strictly derived from racemic ketamine data.

This dose–response pattern highlights a critical duality in ketamine’s pharmacology for obstetric anesthesia. Ultra-low doses (≈0.12 mg/kg) appear to provide a clinically meaningful and relatively safe reduction in spinal anesthesia-induced pruritus. In contrast, the pursuit of effective shivering prophylaxis poses a therapeutic dilemma: the ED_50_ for shivering reduction (≈0.33 mg/kg) exceeds the threshold at which neuropsychiatric adverse effects begin to emerge (≈0.27 mg/kg). Accordingly, ketamine use should be limited to ultra-low doses for pruritus management in cases where alternatives are unsuitable, and patients should be counseled regarding potential (though relatively low-risk) side effects. For shivering, agents with more favorable safety profiles—or non-pharmacological strategies—are preferred. Future research should focus on synergistic low-dose ketamine combinations that may provide anti-shivering benefits without surpassing the adverse effect threshold.

Our findings also demonstrated that ketamine significantly increased the risk of hallucinations, nystagmus, headache, dizziness, and diplopia during cesarean delivery under spinal anesthesia. These adverse outcomes are consistent with ketamine’s mechanism of action through N-methyl-D-aspartate receptor (NMDAR) antagonism (Rolland et al., 2014; Moghaddam and Javitt, 2012). Our results align with a 2024 meta-analysis by Li et al. (2024), which examined ketamine/esketamine for postpartum depression and similarly found higher incidences of dizziness, diplopia, hallucinations, and headache in the ketamine group compared with controls. Notably, both our study and Li et al. (2024)’s analysis revealed considerable heterogeneity in dizziness outcomes (*I* ^2^ = 83%), which was not resolved by sensitivity and subgroup analyses. As such, the dose-response relationship for dizziness should be interpreted with caution, highlighting the need for further research to identify underlying sources of variability.

Conversely, ketamine at 0.329 mg/kg (95%CI, 0.260–0.412) effectively halved the incidence of shivering in our analysis. Zhou et al. (2019) reported no significant benefit for shivering in cesarean sections, though their findings were limited by small sample size (two studies, n = 160) and high heterogeneity (*I* ^2^ = 92%). Mechanistically, ketamine may attenuate shivering either via hypothalamic modulation or through non-shivering thermogenesis mediated by epinephrine’s β-adrenergic activity (Ameta et al., 2018). Clinical evidence supports this hypothesis: Thangavelu et al. (2020) demonstrated that low-dose ketamine reduced shivering incidence despite similar temperature declines in both groups, suggesting a central effect through reduction of the hypothalamic shivering threshold. Similarly, Shakya et al. (2010) reported that ketamine maintained body temperature better than ondansetron or saline, an effect attributed to its vasoconstrictive properties that limit heat redistribution. Beyond peripheral actions, ketamine is also thought to regulate thermogenesis centrally at the hypothalamus and locus coeruleus (Lavelle and MacIomhair, 1975; Canini et al., 2002), further explaining its anti-shivering potential.

Notably, ketamine was effective in preventing pruritus at low doses. This preventive effect is NMDAR dependent. NMDAR is widely distributed in the whole central and peripheral nervous system, and participates in the sensory processing and transmission of pain and pruritus (Petrenko et al., 2014). This phenomenon we found is highly consistent with the molecular mechanism study of Shen et al. (2018) which confirms that intrathecal morphine induces pruritus through activation of NMDA receptors and their downstream ERK phosphorylation pathway in the spinal dorsal horn, which is effectively inhibited by NMDA receptor antagonists such as ketamine and ifenprodil, thereby reducing pruritus behavior (Shen et al., 2018). Our study further supports the value of NMDA receptors as a novel target for pruritus treatment: low-dose ketamine by selectively blocking the spinal cord pruritus signaling pathway.

Additionally, variability in intrathecal adjuvant regimens among included trials complicates interpretation. Notably, among four studies reporting pruritus, Zangouei et al. (2019b) incorporated fentanyl-a potent pruritogen (Ganesh and Maxwell, 2007)-which may have confounded ketamine’s antipruritic effect. Paradoxically, this reinforces our conclusion: despite a baseline pruritus incidence of 50% in the fentanyl-containing study, ketamine (0.15 mg/kg) still reduced the rate to 18.8%, indicating strong intrinsic antipruritic activity. In contrast, in trials without concomitant opioids (e.g., Archana Rani and S. Sen), pruritus incidence was only 0%–8.3%. Thus the evidence presented in this meta-analysis consistently points toward the conclusion that ketamine is effective in preventing intrathecal anesthesia-associated pruritus.

In this analysis, we distinctly categorized and examined three modes of outcome reporting: nausea alone, vomiting alone, and the composite outcome nausea/vomiting. While some studies reported these endpoints separately (Kose et al., 2013; Liu et al., 2023), others used the composite reporting (Ren et al., 2025; Adhikari et al., 2021). It should be noted that this variation in reporting constitutes a potential source of heterogeneity and reporting bias, which may affect the direct comparability and pooled interpretation of results across studies. When analyzed as a composite outcome, the incidence of nausea/vomiting was significantly lower in the ketamine group compared to placebo, with low heterogeneity. However, when nausea and vomiting were assessed as separate endpoints, neither showed a statistically significant difference between the ketamine (0.25 mg/kg) and placebo groups. This finding differs from the study by Li et al. (2024), which reported no between-group difference for nausea/vomiting but noted substantial heterogeneity (*I* ^2^ = 58%); differences in outcome reporting may be one methodological factor contributing to this discrepancy. In our data processing, we did not simply merge the event counts of nausea and vomiting numerically. Further subgroup analysis revealed notable regional heterogeneity in vomiting outcomes: ketamine increased the risk of vomiting in Chinese populations (OR = 3.69, P = 0.02) but decreased the risk in non-Chinese populations (OR = 0.42, P = 0.006), with no significant heterogeneity within each subgroup.

This divergence likely reflects population-specific differences in ketamine metabolism mediated by genetic variations in CYP2B6 allele frequencies, affecting its antiemetic efficacy. East Asian populations (including China) have a significantly lower frequency of the CYP2B6*6 allele and a higher frequency of the CYP2B6*1 allele (Guan et al., 2006). Conversely, in West Asian populations (e.g., Iran, represented in our study), higher CYP2B6*6 and lower CYP2B6*1 frequencies lead to slower ketamine metabolism (Hashemi-Soteh et al., 2021). CYP2B6*1/*1 genotype metabolises ketamine at least twice as fast as CYP2B6*1/*6 and six times faster than CYP2B6*6/*6 (Li et al., 2013). This likely results in faster ketamine metabolism, diminishing its inhibitory effect on central vomiting pathways in East Asian populations.

This study first confirmed the significant association between ketamine and various adverse outcomes (with both triggering and preventive effects) through traditional meta-analysis, but excluded nausea and vomiting (which did not show statistical differences) as the endpoints for subsequent dose-response analysis. The most important central nervous system effect of ketamine is its psychotropic or hallucinogenic effect (Coull et al., 2011; Pomarol-Clotet et al., 2006; Bowdle et al., 1998), and these hallucinogenic side effects are dose-dependent, even occurring at relatively low doses (Niesters et al., 2014). The probability ranking results of the network meta-analysis strongly suggest a potential dose-response relationship between the key adverse outcomes and the dose of ketamine, which has driven the development of the logistic regression model, successfully quantifying the quantitative relationship between the incidence of specific adverse outcomes and the dose of ketamine (0–1 mg/kg).

To overcome the limitation of traditional randomized controlled trials that are difficult to capture the critical risk transition intervals due to the discrete dose grouping, the study constructed a super-large-scale virtual clinical trial (n = 1,001,000) based on the above dose-response model, and precisely simulated the outcome distribution after administration with a step size of 0.001 mg/kg. Combining Monte Carlo simulation and high-precision polynomial regression (R^2^ ≈ 0.99), the study calculated the 95% risk dose (0.761 mg/kg, 95%CI, 0.669–0.880) and the 50% risk dose (0.273 mg/kg, 95%CI, 0.223–0.314) for any subjective discomfort symptoms (such as hallucinations, diplopia, dizziness, headache or drowsiness). The results of the Monte Carlo simulation are highly dependent on the goodness-of-fit of the underlying logistic regression model and the quality of the original studies. If the dose-response relationships in the original studies themselves carry uncertainty, the precision of the simulated ED_50_/ED_95_ values will likewise be affected. The above results were verified through probability sampling, and polynomial regression further optimized the fitting accuracy of the dose-risk curve, significantly improving the stability and generalization ability of threshold prediction.

More sophisticated analytical approaches, such as Bayesian hierarchical dose-response network meta-analysis, were not employed in this study. This decision was driven by two primary considerations. First, the limited number of eligible studies and the relatively small sample sizes within individual dose groups could have rendered a parameter-rich Bayesian hierarchical model unstable or excessively sensitive to prior specifications under conditions of data sparsity. Second, our analysis was explicitly focused on characterizing the dose-response relationship of a single drug class (ketamine/esketamine), rather than conducting a comparative network analysis across multiple agents. Consequently, a conventional dose–response meta-analysis supplemented by simulation was considered more appropriate and methodologically robust for the research objective.

### Considerations on esketamine and dose conversion

It is pertinent to clarify that all studies contributing data to our primary dose-response analysis for shivering and pruritus utilized racemic ketamine. The dose-conversion factor (esketamine dose × 2) outlined in the Methods was a pre-specified rule for evidence synthesis, ensuring a consistent reporting framework should such data be encountered. Nonetheless, the very need for this predefined rule highlights a gap in the current literature: the comparative potency of ketamine versus esketamine for the prophylaxis of anesthesia-related side effects remains formally unestablished, and pharmacological differences between the racemate and the S-enantiomer may exist. Future research directly comparing these agents for specific endpoints is warranted to refine dose recommendations.

### Limitations

This study has several limitations. First, while sensitivity analyses reduced heterogeneity for some outcomes, substantial heterogeneity persisted for estimates of dizziness. Second, the reliability of our findings depends on the quality of the original studies and the goodness of model fit; biases in primary data or model misspecification could affect the precision of ED_50_/ED_95_ estimates. Third, the use of study-level aggregated data instead of individual patient data carries a risk of ecological fallacy. Fourth, the geographic distribution of included studies was imbalanced (approximately 80% from Asia), with insufficient data from other regions to support separate subgroup analyses or dose-response modeling. Fifth, the dose-conversion rule applied to esketamine, while based on analgesic potency, has not been validated for the prophylactic endpoints examined here, representing a methodological constraint. Sixth, the literature search was restricted to three major databases, which may have introduced selection and publication bias by excluding relevant trials indexed in regional databases or clinical trial registries. In addition, the estimated ED_50_ and ED_95_ values represent statistical approximations derived from aggregated data under specific model assumptions. These estimates should therefore be interpreted as providing preliminary guidance for clinical dose selection, rather than definitive dosing recommendations. These limitations highlight the need for future high-quality, multinational RCTs to validate and refine these findings.

## Conclusion

Using Monte Carlo simulation and polynomial regression modeling, this study reconstructed a continuous dose–response profile of racemic ketamine administered during cesarean delivery under spinal anesthesia. Based on current data, the estimated ED_50_ values for racemic ketamine were 0.122 mg/kg for the prevention of pruritus and 0.329 mg/kg for the reduction of shivering. Notably, based on an analysis restricted to studies of racemic ketamine, neuropsychiatric adverse effects emerged at doses as low as 0.273 mg/kg (affecting 50% of patients) and becamed nearly universal at 0.761 mg/kg (affecting 95% of patients). These findings suggested that while low-dose racemic ketamine might be effective for pruritus prevention, its utility for shivering prophylaxis was limited by dose-dependent neuropsychiatric side effects. Careful dose titration and further exploration of alternative approaches were warranted to optimize maternal comfort and safety.

## Data Availability

The original contributions presented in the study are included in the article/Supplementary Material, further inquiries can be directed to the corresponding authors.

## Supplementary material

SUPPLEMENTARY FIGURE S1 Similarity test for network meta-analysis. A, age similarity; B, BMI similarity; C. Gestational age similarity.

SUPPLEMENTARY FIGURE S2 Forest plots of 0.15 mg/kg ketamine versus placebo. A, Pruritus; B, Nausea; C, Headache.

SUPPLEMENTARY FIGURE S3 Forest plots of 0.25 mg/kg ketamine versus placebo. A, nystagmus; B, hallucinations; C, drowsiness; D, dizziness; E, headache; F, hypotension; G, shivering; H, nausea or vomiting; I, nausea; J, vomiting.

SUPPLEMENTARY FIGURE S4 Forest plots of 0.40 mg/kg ketamine versus placebo for nausea/vomiting.

SUPPLEMENTARY FIGURE S5 Forest plots of 0.50 mg/kg ketamine versus placebo. A, nystagmus; B, hallucinations; C, dizziness; D, nausea; E, vomiting.

SUPPLEMENTARY FIGURE S6 Subgroup analysis of 0.25 mg/kg ketamine vomiting with different nations.

SUPPLEMENTARY FIGURE S7 Dose-Response curves of Logistic Regression.

SUPPLEMENTARY TABLE S1 PRISMA 2020 Checklist.

SUPPLEMENTARY TABLE S2 Search syntax for each database.
