# Supplementary material for: Estimated preventive dose of racemic ketamine for shivering and pruritus prophylaxis in cesarean delivery: a Monte Carlo simulation guided network meta-analysis
Source: Front Pharmacol. 2026 Feb 4;17:1751842. doi: 10.3389/fphar.2026.1751842 (PMC12913502; doi:10.3389/fphar.2026.1751842)
Supplement: Supplementary file 1 [file Table4.doc]

Supplemental Table 4. Subgroup analysis for the 0.50 mg/kg ketamine on dizziness.

| Group | Number of  studies | 0.50 mg/kg ketamine | | Control | | M-H pooled OR | | Heterogeneity | |
| --- | --- | --- | --- | --- | --- | --- | --- | --- | --- |
|  |  | event | Total | event | Total | OR(95%CI) | *P* | I^2^ (%) | *P* |
| **0.50 mg/kg ketamine on dizziness** | | | | | | | | | |
| Total | 5 | 120 | 663 | 19 | 659 | 11.18 (1.46, 85.84) | 0.02 | 89 | <0.001 |
| Type of Intraspinal administration | | | | | | |  |  |  |
| 0.5% Bupivacaine 10 mg | 2 | 11 | 116 | 2 | 115 | 5.04 (1.24, 20.58) | 0.02 | <0.001 | 0.73 |
| Other | 3 | 109 | 547 | 17 | 544 | 23.04 (0.89, 595.46) | 0.06 | 94 | <0.001 |
| Combined Intraspinal opioid | | | | | | | | | |
| Yes | 1 | 18 | 327 | 14 | 327 | 1.30 (0.64, 2.66) | 0.47 | NA | NA |
| No | 3 | 74 | 182 | 2 | 178 | 32.09 (0.59, 1745.39) | 0.09 | 86 | 0.0006 |
| Timing of Ketamine used | | | | | |  |  |  |  |
| After fetal extraction | 4 | 119 | 609 | 19 | 605 | 14.56 (1.43, 148.36) | 0.02 | 92 | <0.001 |
| Before spinal anesthesia | 1 | 1 | 54 | 0 | 54 | 3.06 (0.12, 76.70) | 0.50 | NA | NA |
| Type of actual drug use | | | | | | |  |  |  |
| Esketamine | 3 | 101 | 282 | 5 | 278 | 36.89 (2.55, 532.88) | 0.008 | 85.0 | 0.001 |
| Ketamine | 2 | 19 | 381 | 14 | 381 | 1.36 (0.67, 2.73) | 0.39 | <0.001 | 0.61 |

Total =The number of the total patients; OR =Odds ratio; NA =not available.
