# Supplementary figures and images for: Estimated preventive dose of racemic ketamine for shivering and pruritus prophylaxis in cesarean delivery: a Monte Carlo simulation guided network meta-analysis
Source: Front Pharmacol. 2026 Feb 4;17:1751842. doi: 10.3389/fphar.2026.1751842 (PMC12913502; doi:10.3389/fphar.2026.1751842)

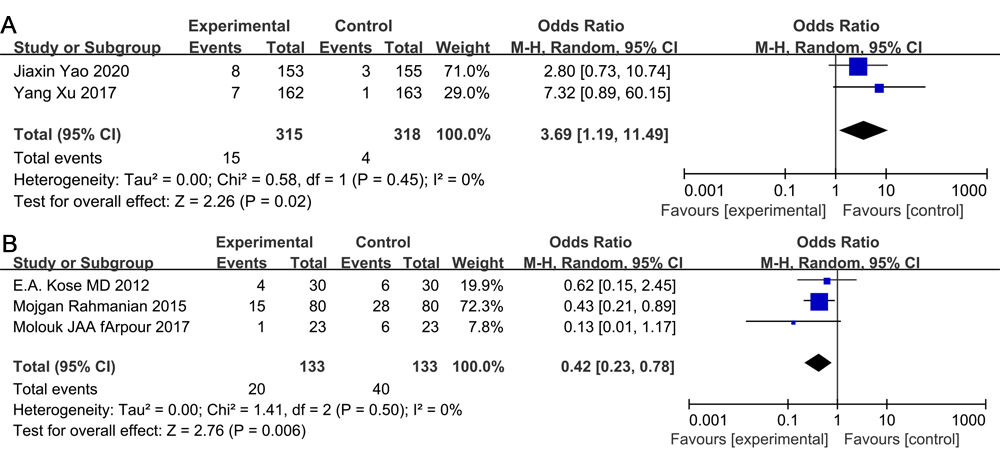

Supplement: Supplementary file 2 [file Image6.tif]

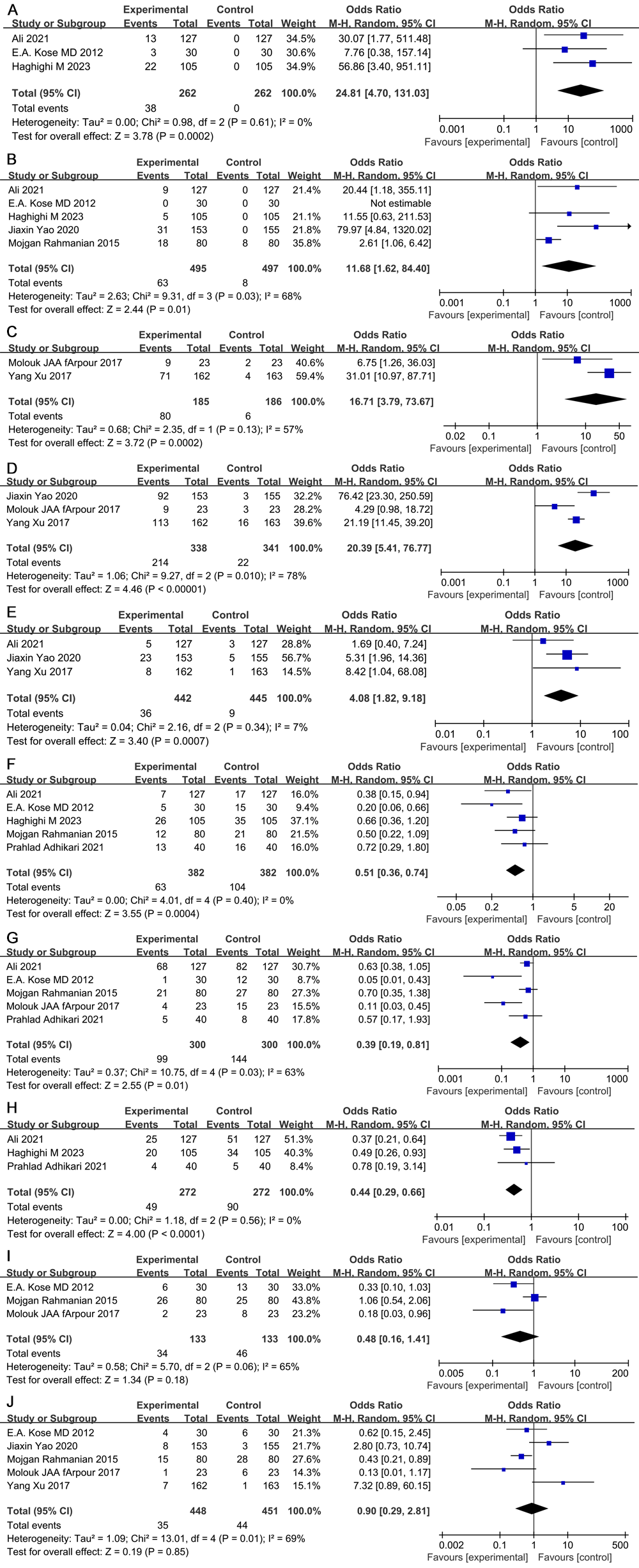

Supplement: Supplementary file 5 [file Image3.tif]

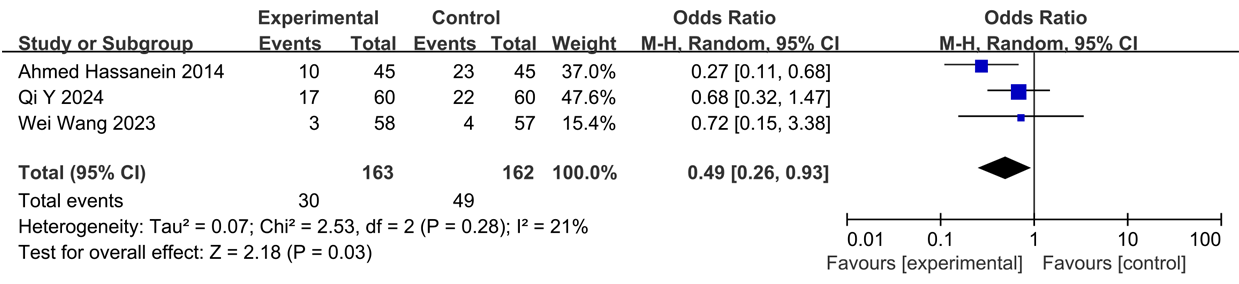

Supplement: Supplementary file 6 [file Image4.tif]

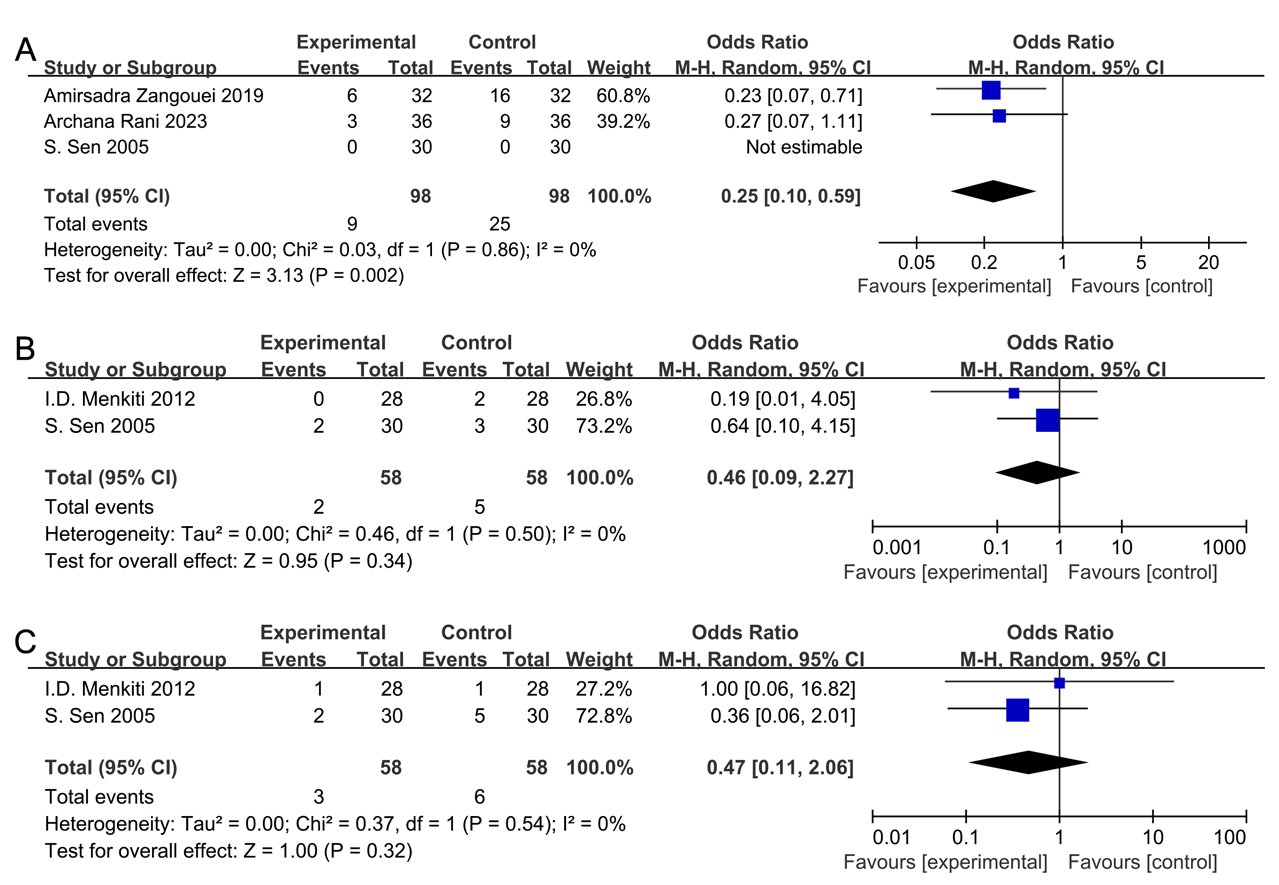

Supplement: Supplementary file 7 [file Image2.tif]

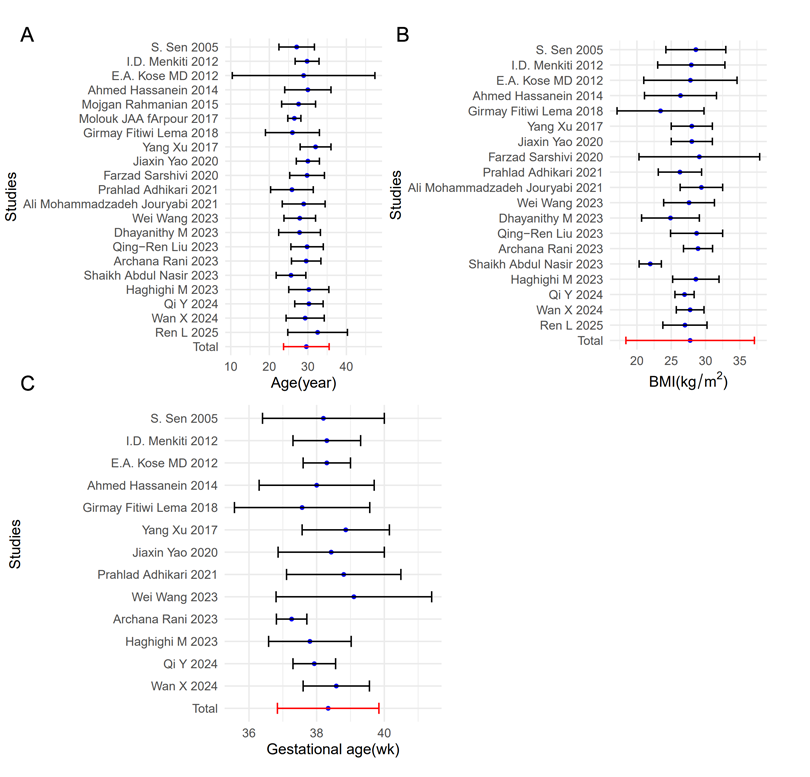

Supplement: Supplementary file 8 [file Image1.tif]

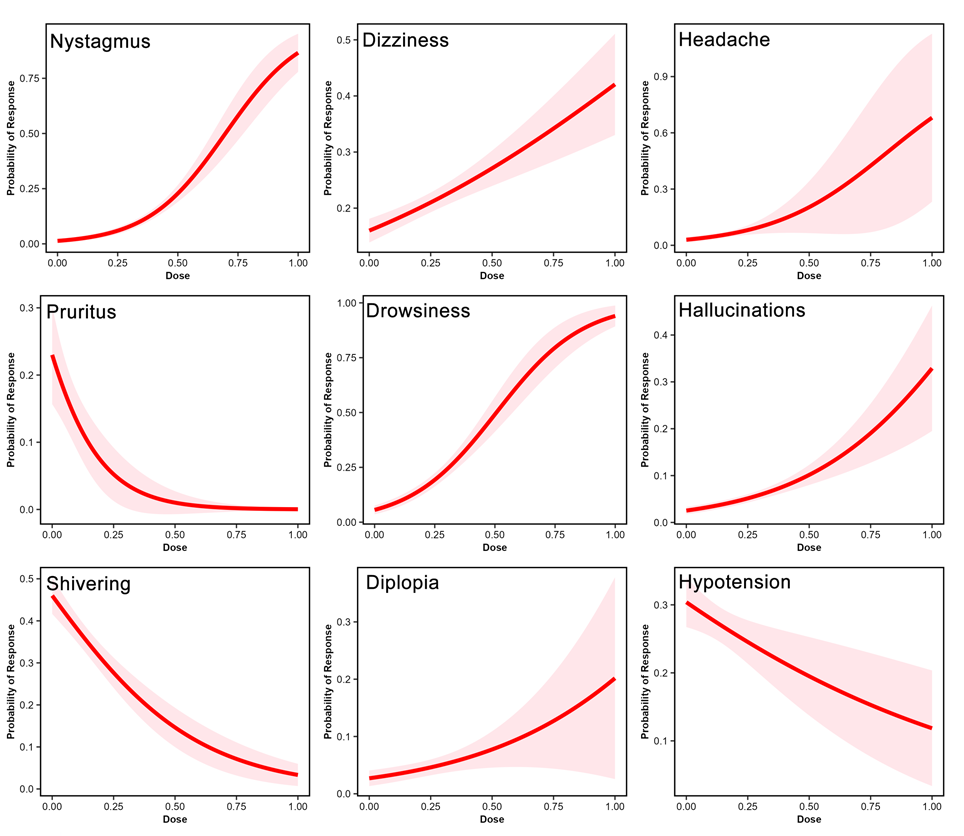

Supplement: Supplementary file 9 [file Image7.tif]

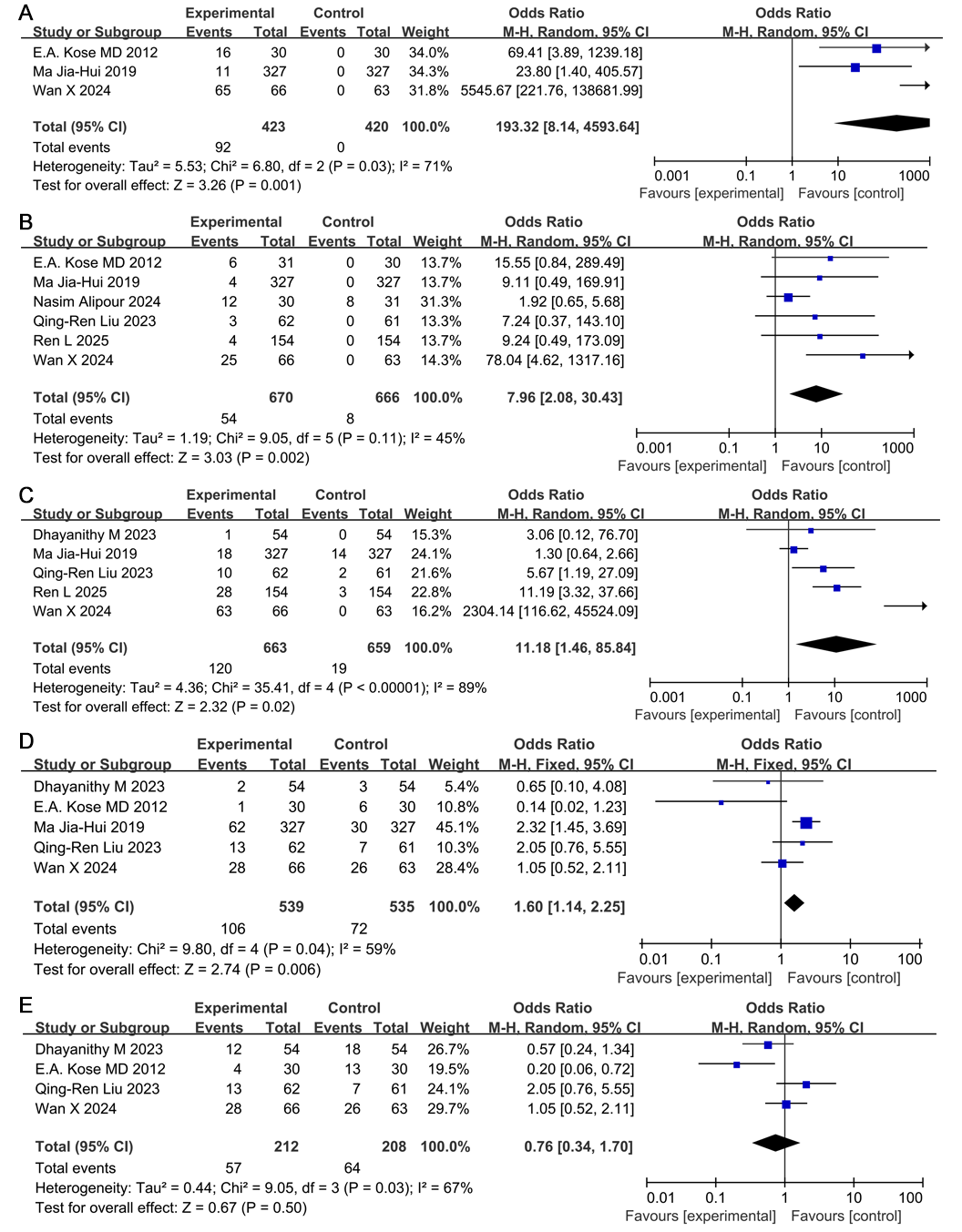

Supplement: Supplementary file 14 [file Image5.tif]
