# Supplementary material for: Estimated preventive dose of racemic ketamine for shivering and pruritus prophylaxis in cesarean delivery: a Monte Carlo simulation guided network meta-analysis
Source: Front Pharmacol. 2026 Feb 4;17:1751842. doi: 10.3389/fphar.2026.1751842 (PMC12913502; doi:10.3389/fphar.2026.1751842)
Supplement: Supplementary file 3 [file Supplementaryfile1.docx]

Supplemental Appendix 1: Definitions for Adverse Events

1. Nystagmus is an involuntary, rapid, rhythmic, oscillatory eye movement with at least 1 slow phase^[1]^.

2. Diplopia or double vision is a condition in which an object is seen as a double image^[2]^.

3. Hallucinations are defined as sensory perceptions that have the compelling sense of reality of true perceptions but that occur without external stimulation of the relevant sensory organ and are experienced as following the sensory path^[3]^.

4. Drowsiness: Subjective desire to sleep or impaired alertness.

5. Dizziness is caused by disturbance of the input or central processing of sensory signals from the vestibular apparatus that provide information regarding the position of the body in space^[4]^.

6. Headache: Pain localized to cranium, orbits, or upper cervical region.

7. Hypotension, is defined as systolic blood pressure <90 mmHg or decrease >20% from baseline^[5]^.

8. Shivering: Oscillatory muscle activity for thermogenesis.

9. Nausea or Vomiting

- Nausea: Subjective urge to vomit.

- Vomiting: Forceful expulsion of gastric contents.

Recorded when any one of above happened.

10. Nausea: As above.

11. Vomiting: As above.

12. Pruritus is defined as an unpleasant sensation resulting in the need to scratch^[6]^.

**References**

[1] Eggers S. Approach to the Examination and Classification of Nystagmus. J Neurol Phys Ther. 2019. 43 Suppl 2: S20-S26.

[2] Jain S. Diplopia: Diagnosis and management. Clin Med (Lond). 2022. 22(2): 104-106.

[3] El-Mallakh RS, Walker KL. Hallucinations, psuedohallucinations, and parahallucinations. Psychiatry. 2010. 73(1): 34-42.

[4] Delaney KA. Bedside diagnosis of vertigo: value of the history and neurological examination. Acad Emerg Med. 2003. 10(12): 1388-95.

[5] Bijker JB, van Klei WA, Kappen TH, van Wolfswinkel L, Moons KG, Kalkman CJ. Incidence of intraoperative hypotension as a function of the chosen definition: literature definitions applied to a retrospective cohort using automated data collection. Anesthesiology. 2007. 107(2): 213-20.

[6] Welz-Kubiak K, Reszke R, Szepietowski JC. Pruritus as a sign of systemic disease. Clin Dermatol. 2019. 37(6): 644-656.
