# Supplementary material for: Estimated preventive dose of racemic ketamine for shivering and pruritus prophylaxis in cesarean delivery: a Monte Carlo simulation guided network meta-analysis
Source: Front Pharmacol. 2026 Feb 4;17:1751842. doi: 10.3389/fphar.2026.1751842 (PMC12913502; doi:10.3389/fphar.2026.1751842)
Supplement: Supplementary file 10 [file Table2.docx]

| **Database** | Pubmed | EMBASE | Cochrane Library |
| --- | --- | --- | --- |
| **Date** | 25 Apr 2025 | 25 Apr 2025 | 25 Apr 2025 |
| **Filters applied** | Randomized Controlled Trial | / | Trial |
| **Records retrieved** | 54 | 164 | 89 |

**Searchfilter---Pubmed**

(("ketamine"[MeSH Terms] OR "esketamine"[Supplementary Concept] OR ((((("2"[All Fields] AND "2-Chlorophenyl"[All Fields]) AND "2"[All Fields]) AND "methylamino"[All Fields]) AND ("cyclohexanone"[Supplementary Concept] OR "cyclohexanone"[All Fields] OR "cyclohexanones"[MeSH Terms] OR "cyclohexanones"[All Fields])) OR (((("2"[All Fields] AND "2-Chlorophenyl"[All Fields]) AND "2"[All Fields]) AND "methylamino"[All Fields]) AND ("cyclohexanone"[Supplementary Concept] OR "cyclohexanone"[All Fields] OR "cyclohexanones"[MeSH Terms] OR "cyclohexanones"[All Fields])) OR ("ketamin"[All Fields] OR "ketamine"[Supplementary Concept] OR "ketamine"[All Fields] OR "esketamine"[Supplementary Concept] OR "esketamine"[All Fields] OR "esketamine"[Supplementary Concept] OR "esketamine"[All Fields] OR "ketamine"[MeSH Terms] OR "ketalar"[All Fields] OR "ketamine s"[All Fields] OR "ketamines"[All Fields]) OR ("ketamine"[Supplementary Concept] OR "ketamine"[All Fields] OR "ci 581"[All Fields] OR "ketamine"[MeSH Terms]) OR ("ketamine"[Supplementary Concept] OR "ketamine"[All Fields] OR "ci581"[All Fields] OR "ketamine"[MeSH Terms]) OR ("ketamine"[Supplementary Concept] OR "ketamine"[All Fields] OR "ci 581"[All Fields] OR "ketamine"[MeSH Terms]) OR ("ketamine"[Supplementary Concept] OR "ketamine"[All Fields] OR "ketanest"[All Fields] OR "ketamine"[MeSH Terms]) OR ("ketamine"[Supplementary Concept] OR "ketamine"[All Fields] OR "ketamine hydrochloride"[All Fields] OR "ketamine"[MeSH Terms] OR ("ketamine"[All Fields] AND "hydrochloride"[All Fields])) OR ("ketamine"[Supplementary Concept] OR "ketamine"[All Fields] OR "calipsol"[All Fields] OR "ketamine"[MeSH Terms]) OR ("ketamine"[Supplementary Concept] OR "ketamine"[All Fields] OR "calypsol"[All Fields] OR "ketamine"[MeSH Terms]) OR ("ketamine"[Supplementary Concept] OR "ketamine"[All Fields] OR "kalipsol"[All Fields] OR "ketamine"[MeSH Terms]) OR ("ketamin"[All Fields] OR "ketamine"[Supplementary Concept] OR "ketamine"[All Fields] OR "esketamine"[Supplementary Concept] OR "esketamine"[All Fields] OR "esketamine"[Supplementary Concept] OR "esketamine"[All Fields] OR "ketamine"[MeSH Terms] OR "ketaset"[All Fields] OR "ketamine s"[All Fields] OR "ketamines"[All Fields])) OR ("esketamine"[Supplementary Concept] OR "esketamine"[All Fields] OR "l ketamine"[All Fields] OR ("ketamin"[All Fields] OR "ketamine"[Supplementary Concept] OR "ketamine"[All Fields] OR "esketamine"[Supplementary Concept] OR "esketamine"[All Fields] OR "esketamine"[Supplementary Concept] OR "esketamine"[All Fields] OR "ketamine"[MeSH Terms] OR "ketamine s"[All Fields] OR "ketamines"[All Fields]) OR ("esketamine"[Supplementary Concept] OR "esketamine"[All Fields] OR "s ketamine"[All Fields]) OR ((((("S"[All Fields] AND "2"[All Fields]) AND "o-chlorophenyl"[All Fields]) AND "2"[All Fields]) AND "methylamino"[All Fields]) AND ("cyclohexanone"[Supplementary Concept] OR "cyclohexanone"[All Fields] OR "cyclohexanones"[MeSH Terms] OR "cyclohexanones"[All Fields])) OR ("esketamine"[Supplementary Concept] OR "esketamine"[All Fields]) OR ("esketamine"[Supplementary Concept] OR "esketamine"[All Fields] OR "spravato"[All Fields]))) AND ("cesarean section"[MeSH Terms] OR ("caesarean sections"[All Fields] OR "cesarean section"[MeSH Terms] OR ("cesarean"[All Fields] AND "section"[All Fields]) OR "cesarean section"[All Fields] OR ("cesarean"[All Fields] AND "sections"[All Fields]) OR "cesarean sections"[All Fields]) OR ("cesarean section"[MeSH Terms] OR ("cesarean"[All Fields] AND "section"[All Fields]) OR "cesarean section"[All Fields] OR ("abdominal"[All Fields] AND "delivery"[All Fields]) OR "abdominal delivery"[All Fields]) OR (("cesarean section"[MeSH Terms] OR ("cesarean"[All Fields] AND "section"[All Fields]) OR "cesarean section"[All Fields] OR "c section"[All Fields]) AND "OB"[All Fields]) OR (("cesarean section"[MeSH Terms] OR ("cesarean"[All Fields] AND "section"[All Fields]) OR "cesarean section"[All Fields] OR "c section"[All Fields]) AND "OB"[All Fields]) OR (("cesarean section"[MeSH Terms] OR ("cesarean"[All Fields] AND "section"[All Fields]) OR "cesarean section"[All Fields] OR "c sections"[All Fields]) AND "OB"[All Fields]) OR ("caesarean section"[All Fields] OR "cesarean section"[MeSH Terms] OR ("cesarean"[All Fields] AND "section"[All Fields]) OR "cesarean section"[All Fields]) OR ("caesarean sections"[All Fields] OR "cesarean section"[MeSH Terms] OR ("cesarean"[All Fields] AND "section"[All Fields]) OR "cesarean section"[All Fields] OR ("cesarean"[All Fields] AND "sections"[All Fields]) OR "cesarean sections"[All Fields]) OR ("cesarean section"[MeSH Terms] OR ("cesarean"[All Fields] AND "section"[All Fields]) OR "cesarean section"[All Fields] OR ("delivery"[All Fields] AND "abdominal"[All Fields]) OR "delivery abdominal"[All Fields]) OR ("cesarean section"[MeSH Terms] OR ("cesarean"[All Fields] AND "section"[All Fields]) OR "cesarean section"[All Fields] OR ("abdominal"[All Fields] AND "deliveries"[All Fields]) OR "abdominal deliveries"[All Fields]) OR ("cesarean section"[MeSH Terms] OR ("cesarean"[All Fields] AND "section"[All Fields]) OR "cesarean section"[All Fields] OR ("deliveries"[All Fields] AND "abdominal"[All Fields]) OR "deliveries abdominal"[All Fields]) OR ("cesarean section"[MeSH Terms] OR ("cesarean"[All Fields] AND "section"[All Fields]) OR "cesarean section"[All Fields] OR ("postcesarean"[All Fields] AND "section"[All Fields]) OR "postcesarean section"[All Fields]))) AND (randomizedcontrolledtrial[Filter])

**Searchfilter---EMBASE**

#1 'ketamine'/exp
#2 '2 (2 chlorophenyl) 2 (methylamino) cyclohexanone' OR '2 (2 chlorophenyl) 2 (methylamino) cyclohexanone hydrochloride' OR '2 (2 chlorophenyl) 2 methylaminocyclohexanone' OR '2 (methylamino) 2 (2 chlorophenyl) cyclohexanone' OR '2 (ortho chlorophenyl) 2 (methylamino) cyclohexanone' OR '2 (ortho chlorophenyl) 2 methylaminocyclohexanone' OR '2 (ortho chlorophenyl) 2 methylaminocyclohexanone hydrochloride' OR '2 methylamino 2 (2 chlorophenyl) cyclohexanone' OR '2 ortho chlorophenyl 2 methylaminocyclohexanone' OR 'anesject' OR 'calipsol' OR 'calypsol' OR 'ci 581' OR 'ci581' OR 'cl 369' OR 'cl369' OR 'cn 52, 372 2' OR 'cn 52372 2' OR 'cn 523722' OR 'cn52, 372 2' OR 'cn52372 2' OR 'cn523722' OR 'ereska' OR 'imalgene' OR 'kalipsol' OR 'katamine' OR 'keta-hameln' OR 'ketaject' OR 'ketalar' OR 'ketalin' OR 'ketamax' OR 'ketamine hcl' OR 'ketamine hydrochloride' OR 'ketaminol vet' OR 'ketanest' OR 'ketased' OR 'ketaset' OR 'ketaved' OR 'ketavet' OR 'ketmin' OR 'ketoject' OR 'ketolar' OR 'narkamon' OR 'narketan' OR 'pmi 100' OR 'pmi 150' OR 'pmi100' OR 'pmi150' OR 'sls 002' OR 'sls002' OR 'soon-soon' OR 'tekam' OR 'tur 002' OR 'tur002' OR 'velonarcon' OR 'vetalar' OR 'ketamine'

#3 #1 OR #2
#4 'cesarean section'/exp
#5 'birth, abdominal operation' OR 'birth, caesarean' OR 'caesarean birth' OR 'caesarean section' OR 'caesarian birth' OR 'caesarian section' OR 'cesarean delivery' OR 'cesarian section' OR 'fetectomy' OR 'repeated cesarotomy' OR 'sectio caesarea' OR 'cesarean section'

#6 #4 OR #5

#7 #2 AND #6

#8 'randomized controlled trial'/exp

#9 'controlled trial, randomized' OR 'randomised controlled study' OR 'randomised controlled trial' OR 'randomized controlled study' OR 'trial, randomized controlled' OR 'randomized controlled trial'

#10 #8 OR #9

#11 #7 AND #10

**Searchfilter---Cochrane Library**

#1 MeSH descriptor: [Ketamine] explode all trees

#2 (Ketalar) (Word variations have been searched)

#3 (CI-581) (Word variations have been searched)

#4 (CI581) (Word variations have been searched)

#5 (CI 581) (Word variations have been searched)

#6 (Ketanest) (Word variations have been searched)

#7 (Ketamine Hydrochloride) (Word variations have been searched)

#8 (Calipsol) (Word variations have been searched)

#9 (Calypsol) (Word variations have been searched)

#10 (Kalipsol) (Word variations have been searched)

#11 (Ketaset) (Word variations have been searched)

#12 #1 OR #2 OR #3 OR #4 OR #5 OR #6 OR #7 OR #8 OR #9 OR #10 OR #11

#13 MeSH descriptor: [Cesarean Section] explode all trees

#14 (Cesarean Sections) (Word variations have been searched)

#15 (Abdominal Delivery) (Word variations have been searched)

#16 (C-Section (OB)) (Word variations have been searched)

#17 (C Section (OB)) (Word variations have been searched)

#18 (C-Sections (OB)) (Word variations have been searched)

#19 (Caesarean Section) (Word variations have been searched)

#20 (Caesarean Sections) (Word variations have been searched)

#21 (Delivery, Abdominal) (Word variations have been searched)

#22 (Abdominal Deliveries) (Word variations have been searched)

#23 (Deliveries, Abdominal) (Word variations have been searched)

#24 (Postcesarean Section) (Word variations have been searched)

#25 #13 OR #14 OR #15 OR #16 OR #17 OR #18 OR #19 OR #20 OR #21 OR #22 OR #23 OR #24

#26 #12 AND #25
