# Supplementary material for: Estimated preventive dose of racemic ketamine for shivering and pruritus prophylaxis in cesarean delivery: a Monte Carlo simulation guided network meta-analysis
Source: Front Pharmacol. 2026 Feb 4;17:1751842. doi: 10.3389/fphar.2026.1751842 (PMC12913502; doi:10.3389/fphar.2026.1751842)
Supplement: Supplementary file 11 [file Table5.docx]

| **Supplemental Table 5 \| Comparison of Direct and Indirect Evidence Odds Ratios(ORs) and Ratio of ORs(ROR)** | | | | |
| --- | --- | --- | --- | --- |
| Adverse Event | Direct Evidence OR(95%CI) | Indirect Evidence OR(95%CI) | ROR(95%CI) | Consistency  (P>0.05) |
| Hypotension | 2.80(0.50, 15.73) | 5.14(1.08, 24.53) | 0.54(0.05, 5.58) | Yes |
| Nausea | 1.62(0.26, 10.18) | 0.77(0.25, 2.37) | 2.10(0.24, 18.08) | Yes |
| Shiver | 3.10(0.10, 96.77) | 3.54(0.46, 27.16) | 0.88(0.02, 47.66) | Yes |
| Hallucination | 0.06(0.00, 2.50) | 0.63(0.10, 4.05) | 0.10(0.00, Inf) | Yes |
| Vomiting | 4.46(0.30, 67.38) | 0.91(0.26, 3.12) | 4.90(0.25, 96.36) | Yes |
| Itching | 3.18(0.32, 32.14) | 3.10(0.35, 27.28) | 1.03(0.04, 24.45) | Yes |
| Nystagmus | 0.11(0.01, 1.81) | 0.12(0.01, 1.18) | 0.92(0.03, 31.21) | Yes |
