# Supplementary material for: Estimated preventive dose of racemic ketamine for shivering and pruritus prophylaxis in cesarean delivery: a Monte Carlo simulation guided network meta-analysis
Source: Front Pharmacol. 2026 Feb 4;17:1751842. doi: 10.3389/fphar.2026.1751842 (PMC12913502; doi:10.3389/fphar.2026.1751842)
Supplement: Supplementary file 12 [file Table3.docx]

| Supplemental Table 3. Sensitivity Analysis for Ketamine versus Placebo | | | | | |
| --- | --- | --- | --- | --- | --- |
| Study omitted | M-H pooled OR | | Heterogeneity | | Robustness  (yes/no/unclear) |
|  | OR (95%CI) | *P* value | *I*^2^ (%) | *P* value |  |
| 0.25mg/kg |  |  |  |  |  |
| Hallucination | 11.68 (1.62, 84.40) | 0.01 | 68 | 0.03 | yes |
| Ali 2021 | 10.75 (0.85, 135.83) | 0.07 | 75 | 0.02 |  |
| E.A. Kose MD 2012 | 11.68 (1.62, 84.40) | 0.01 | 68 | 0.03 |  |
| Haghighi M 2023 | 12.97 (0.91, 184.80) | 0.06 | 78 | 0.01 |  |
| Jiaxin Yao 2020 | 4.84 (1.28, 18.29) | 0.02 | 29 | 0.25 |  |
| Mojgan Rahmanian 2015 | 27.26 (5.25, 141.68) | <0.0001 | <0.0001 | 0.60 |  |
| Dizziness* | 20.39 (5.41, 76.77) | <0.0001 | 78 | 0.01 | unclear |
| Jiaxin Yao 2020 | 11.03 (2.36, 51.44) | 0.002 | 74 | 0.05 |  |
| Molouk JAA fArpour 2017 | 36.51 (10.06, 132.43) | <0.0001 | 74 | 0.05 |  |
| Yang Xu 2017 | 18.71 (1.05, 333.23) | 0.05 | 89 | 0.002 |  |
| Shivering | 0.39 (0.19, 0.81) | 0.01 | 63 | 0.03 | yes |
| Ali 2021 | 0.28 (0.09, 0.86) | 0.03 | 69 | 0.02 |  |
| E.A. Kose MD 2012 | 0.51 (0.28, 0.91) | 0.02 | 49 | 0.12 |  |
| Mojgan Rahmanian 2015 | 0.28 (0.10, 0.82) | 0.02 | 70 | 0.02 |  |
| Molouk JAA fArpour 2017 | 0.54 (0.29, 0.98) | 0.04 | 45 | 0.14 |  |
| Prahlad Adhikari 2021 | 0.34 (0.14, 0.83) | 0.02 | 72 | 0.01 |  |
| Nausea | 0.48 (0.16, 1.41) | 0.18 | 65 | 0.06 | no |
| E.A. Kose MD 2012 | 0.52 (0.09, 2.88) | 0.45 | 73 | 0.05 |  |
| Mojgan Rahmanian 2015 | 0.27 (0.10, 0.70) | 0.007 | <0.0001 | 0.56 |  |
| Molouk JAA fArpour 2017 | 0.65 (0.21, 2.02) | 0.46 | 67 | 0.08 |  |
| Vomiting* | 0.90 (0.29, 2.81) | 0.85 | 69 | 0.01 | unclear |
| E.A. Kose MD 2012 | 1.01 (0.22, 4.63) | 0.99 | 77 | 0.005 |  |
| Jiaxin Yao 2020 | 0.65 (0.19, 2.18) | 0.48 | 63 | 0.04 |  |
| Mojgan Rahmanian 2015 | 1.18 (0.27, 5.22) | 0.83 | 67 | 0.03 |  |
| Molouk JAA fArpour 2017 | 1.24 (0.36, 4.22) | 0.73 | 72 | 0.01 |  |
| Yang Xu 2017 | 0.63 (0.22, 1.79) | 0.38 | 61 | 0.05 |  |
| 0.50mg/kg |  |  |  |  |  |
| Nystagmus | 193.32 (8.14, 4593.64) | 0.001 | 71 | 0.03 | yes |
| E.A. Kose MD 2012 | 344.40 (1.49, 79532.07) | 0.04 | 84 | 0.01 |  |
| Ma Jia-Hui 2019 | 584.86 (7.44, 46006.63) | 0.004 | 76 | 0.04 |  |
| Wan X 2024 | 40.29 (5.34, 304.14) | 0.0003 | <0.0001 | 0.60 |  |
| Dizziness* | 11.18 (1.46, 85.84) | 0.02 | 89 | <0.0001 | unclear |
| Dhayanithy M 2023 | 14.56 (1.43, 148.36) | 0.02 | 92 | <0.0001 |  |
| Ma Jia-Hui 2019 | 21.74 (2.35, 201.48) | 0.007 | 80 | 0.002 |  |
| Qing-Ren Liu 2023 | 14.59 (0.98, 218.04) | 0.05 | 92 | <0.0001 |  |
| Ren L 2025 | 12.51 (0.68, 231.67) | 0.09 | 91 | <0.0001 |  |
| Wan X 2024 | 3.86 (1.05, 14.22) | 0.04 | 72 | 0.01 |  |
| Nausea | 0.76 (0.34, 1.70) | 0.50 | 65 | 0.03 | yes |
| Dhayanithy M 2023 | 0.82 (0.27, 2.52) | 0.73 | 75 | 0.02 |  |
| E.A. Kose MD 2012 | 1.03 (0.54, 1.99) | 0.93 | 45 | 0.16 |  |
| Qing-Ren Liu 2023 | 0.56 (0.24, 1.31) | 0.19 | 61 | 0.08 |  |
| Wan X 2024 | 0.65 (0.19, 2.18) | 0.48 | 76 | 0.02 |  |
| Vomiting | 1.60 (1.14, 2.25) | 0.006 | 59 | 0.04 | no |
| Dhayanithy M 2023 | 1.66 (1.17, 2.34) | 0.004 | 66 | 0.03 |  |
| E.A. Kose MD 2012 | 1.78 (1.26, 2.53) | 0.001 | 35 | 0.20 |  |
| Ma Jia-Hui 2019 | 1.02 (0.61, 1.69) | 0.94 | 44 | 0.15 |  |
| Qing-Ren Liu 2023 | 1.55 (1.08, 2.23) | 0.02 | 69 | 0.02 |  |
| Wan X 2024 | 1.83 (1.24, 2.69) | 0.002 | 61 | 0.05 |  |
| Note: A significant level of heterogeneity was considered when the *I*^2^ statistic exceeded 50% and the corresponding *P* value for *I*^2^ was less than 0.10.  *For the outcomes of dizziness in both the 0.25 mg/kg and 0.50 mg/kg groups, and vomiting in the 0.25 mg/kg group, no single study was identified as a clear source of heterogeneity. Further subgroup analyses may be helpful to clarify the underlying causes.  Abbreviation: OR, odds ratio; CI,confidence interval | | | | | |
