# Supplementary material for: Estimated preventive dose of racemic ketamine for shivering and pruritus prophylaxis in cesarean delivery: a Monte Carlo simulation guided network meta-analysis
Source: Front Pharmacol. 2026 Feb 4;17:1751842. doi: 10.3389/fphar.2026.1751842 (PMC12913502; doi:10.3389/fphar.2026.1751842)
Supplement: Supplementary file 13 [file Table6.docx]

| Supplemental Table 6 \| Monte Carlo Simulation in Subgroup of Asian | | |
| --- | --- | --- |
| Outcomes | Estimated Effect Dose | Estimated 95%CI |
| Prevention |  |  |
| Pruritus, ED_50_ | 0.128 | 0.089–0.174 |
| Shivering, ED_50_ | 0.413 | 0.302–0.553 |
| Subject discomfort |  |  |
| ED_50_ | 0.270 | 0.230–0.313 |
| ED_95_ | 0.719 | 0.635–0.825 |
| Note: All results are mixed population model-based estimates and are presented as ED_50_ or ED_95_ with doses in milligrams per kilogram of body weight(mg/kg).  Abbreviation: CI, confidence interval. | | |
